# NbN films on flexible and thickness controllable dielectric substrates

**DOI:** 10.1038/s41598-022-14861-z

**Published:** 2022-06-23

**Authors:** Hongkai Shi, Lanju Liang, Yi Huang, Han Bao, Biaobing Jin, Zhihe Wang, Xiaoqing Jia, Lin Kang, Weiwei Xu, Jian Chen, Peiheng Wu

**Affiliations:** 1 Research Institute of Superconductor Electronics, School of Electronic Science and Engineering, Nanjing University, Nanjing, 210093 People’s Republic of China; 2 Purple Mountain Laboratories, Nanjing, 211111 People’s Republic of China; 3 School of Opto-Electronic Engineering, Zao Zhuang University, Zao Zhuang, 277160 People’s Republic of China

**Keywords:** Polymers, Sensors and biosensors, Nanoscale materials

## Abstract

A simple method for preparing superconducting NbN thin films on flexible dielectric substrates with controllable thickness was developed. The structure and surface characteristics and superconducting properties of the flexible film were studied by X-ray diffraction (XRD), atomic force microscopy (AFM) and physical property measurement system (PPMS). We found that NbN films on the flexible substrate show certain preferred orientations through the self-buffering effect of the amorphous NbN layer. The zero resistance superconducting transition temperature (T_C0_) for 10 nm thick NbN films is 8.3 K, and the T_C0_ for 30 nm thick NbN films in a magnetic field of 9 T remains above 7 K. This flexible film can be transferred to any substrate and adapted to different shape applications. It can also be further processed into single-layer or multilayer flexible superconducting devices.

## Introduction

The superconducting niobium nitride (NbN) film has a relatively high superconducting transition temperature (Tc) and high critical current density^1^. Hence, NbN films have been widely utilized in superconducting electronic devices, especially for extremely sensitive detectors such as hot electron bolometers^2, 3^ and superconducting nanowire single photon detectors^4, 5^. Substrates are one of the foundations for NbN film preparation and directly affect the superconductivity of the film and the coupling of devices and electromagnetic waves. NbN is usually prepared on MgO^6^, Al_2_O_3_^7^, GaAs^8^, silicon (Si)^9^ and other substrates. The choice of substrate mainly aims to improve the performance of the device and match the application situation. The lattice mismatch between the substrate and film can also directly affect the performance of the film and device. Buffer layers can be used between the substrate and film^10^ to solve the mismatch to some extent and improve the performance of NbN films and devices. The thickness of the substrate affects the coupling efficiency of the device and the electromagnetic wave signal, especially in the terahertz frequency band where the substrate thickness and electromagnetic wavelength are similar, and an interference effect between the substrate and film can occur^11^. To reduce the interference effect and substrate loss, etching processes can be used to reduce the thickness of the substrate^12–14^. However, the mechanical strength of rigid substrates will be greatly reduced with the thinning of the substrate thickness and become very fragile. Use of flexible substrates can avoid such problems. Moreover, the growth of superconducting films on flexible substrates can also be used in the preparation of multilayer structure devices^15–18^.

Highly conductive materials are often used to shield external magnetic fields, but the frequency of the external magnetic field has to be sufficiently large (> 1 kHz)^19^; the magnetic permeability of conductive materials will be quite poor at low temperatures since the skin depth δ is quite large at low frequencies. At frequencies below 1 kHz and in low-temperature environments, superconductors shield magnetic fields more effectively than ferromagnetic materials because of their strong diamagnetic properties. When a superconducting material is cooled below the phase transition temperature Tc, superconducting materials expel magnetic fields by generating screening currents that oppose the external magnetic field, which is called the Meissner effect. Superconducting films with a high critical current density, sufficient mechanical strength and ductility^19, 20^ are required for efficient magnetic shielding. The effectiveness of the shield also depends on the material quality, microstructure and shape^19^. YBCO^20^, MgB_2_^21, 22^ and other superconducting films have been used in practical applications. MgB_2_ with a critical current density of 30 kA cm^−2^ and critical magnetic field of 12.8 T can achieve a magnetic field shielding of 2 T at 4.2 K^21^.

Since a J_C_ of higher than 10^7^ A cm^−2^ can be obtained for a 6 nm thick NbN film at 4.2 K^10^, NbN films can also have promising applications in magnetic shielding due to their high Tc at small thickness, good mechanical properties, and large superconducting critical magnetic field^23^. Flexible substrates can be combined with ferromagnetic materials to realize the cloaking of magnetic fields^24^, which can simplify the preparation process. At the same time, flexible substrates can be tailored and applied to the corresponding structure, which is more convenient for applications. Free-standing and flexible NbN films several hundred nanometres thick have been realized on graphene substrates; however, the area of such films is still relatively small^25^.

To date, common flexible dielectric substrates include polydimethylsiloxane (PDMS), polyethylene terephthalate (PET), and polyimide (PI)^26^. Among them, the temperature sensitivity of PDMS is quite high (TEC of 3.1 × 10^–4^/°C), which can lead to stress variation in thin films and changes in the geometric structure of devices, which make it unsuitable for low-temperature situations. PET is quite costly, and, thus, is difficult to use widely. PI is a cyclic polymer containing an imide cyclic structure in the main chain of the molecule, which has the widest operating temperature between − 269 and 400 °C and shows good insulation properties (resistivity of 1.7 × 10^17^ Ω·cm), flexibility (Young’s modulus of 2.5 GPa), and low absorption losses (12 cm^−1^ at 1 THz). In this work, we demonstrate a method to prepare a high-performance superconducting film with high flatness on a flexible PI substrate. After further micro/nanofabrication, flexible superconducting devices are prepared, which can also be applied onto nonflat surfaces and other complex occasions, which greatly extends the application and scope for superconducting films and devices.

## Methods

**Figure 1:**
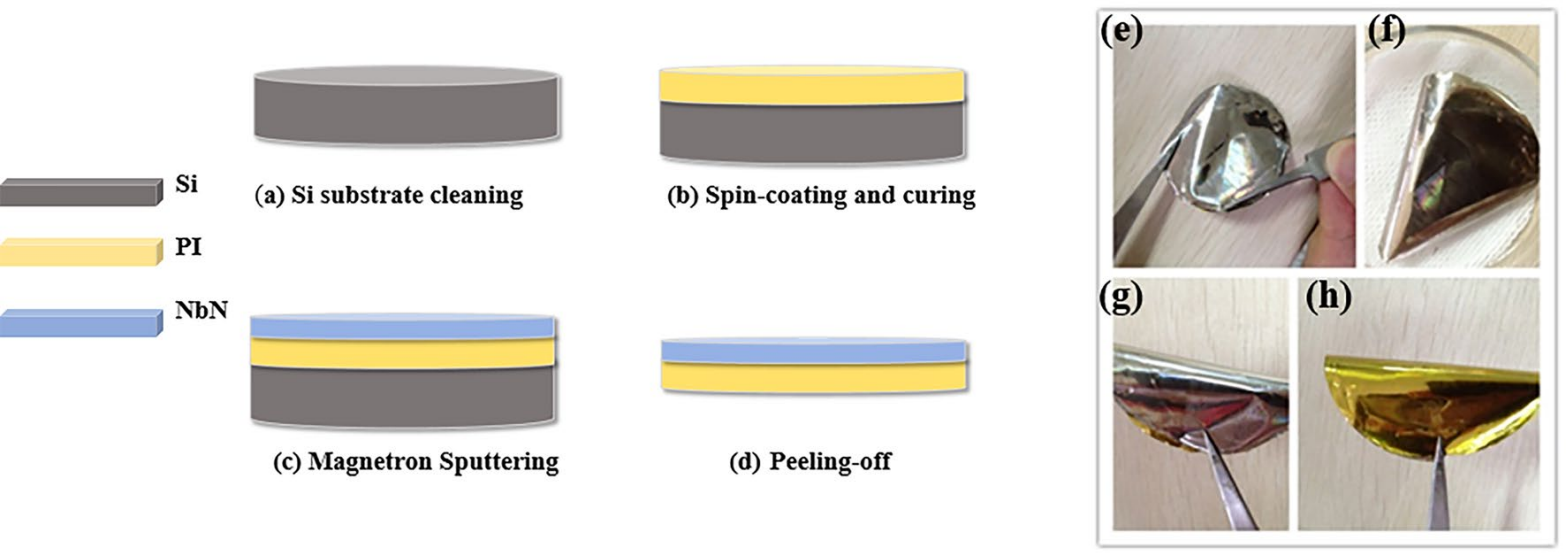
Preparation process (left: **a**–**d**), the prepared 2-inch sample (right: **e**–**h**).

High-resistance silicon is used as the foundation for the preparation of flexible substrates. First, as shown in Fig. 1a, acetone, alcohol, and deionized water are used to clean the Si substrate for 3 min. As a result, organic contaminants on the substrate surface can be removed, and the PI layers can firmly adhere to the substrate. In addition, one can also prevent the formation of pinholes in the PI.

**Figure 2:**
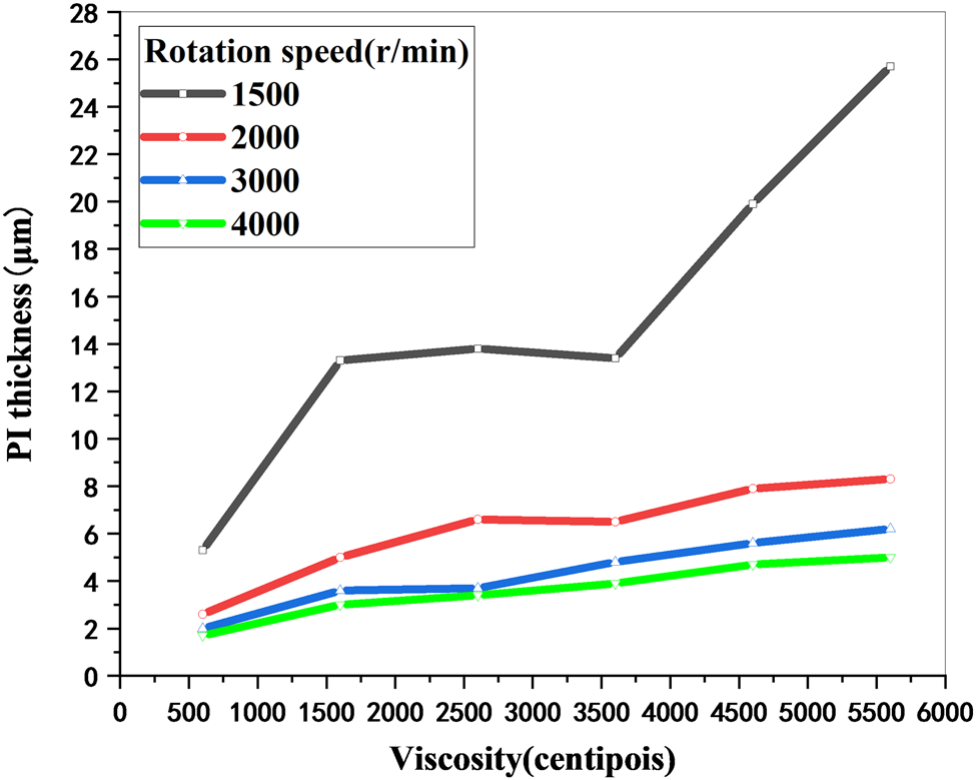
The relationship between the PI layer thickness, rotation speed and PI viscosity.

The thickness of the PI film is mainly determined by the rotational speed and the viscosity of the PI. If the rotation speed is quite low, the thickness of the PI film will not be very uniform. By controlling the PI viscosity, spin coating speed and rotation time, the thickness of the PI film can be precisely controlled, as shown in Fig. 2. For example, to obtain a 4 μm thick PI film, PI with a viscosity of 3600 centipoises can be spin coated onto the substrate at a rotation speed of 4000 r/min for a spin time of 1 min. As shown in Fig. 1b, spin-coated PI was placed into a vacuum drying environment for curing. The curing temperature was 120 °C for 1 h, 200 °C for 1 h, 230 °C for 1 h, and 250 °C for 2 h^27^. Then, the sample was exposed at room temperature and cooled naturally. The number of spin coating cycles and curing cycles can be adjusted according to the thickness requirements of the PI layer. For the case of a thick PI layer, multiple spin coating and curing can be adopted to avoid the formation of air gaps between PI layers and ensure a smooth surface. For lower PI thicknesses, lower viscosities and higher rotational speeds are needed; for example, the lowest thickness of 1 μm can be obtained by spin coating PI with a viscosity of 600 centipoises at a speed of 8000 r/min for 1 min. A thinner PI can be obtained by further diluting the PI solution.

After spin coating, as shown in Fig. 1c, we used DC reactive magnetron sputtering technology to prepare the niobium nitride (NbN) superconducting film on a PI-coated Si substrate. We used a 4-inch diameter niobium sputtering target with a purity of 99.999%. The distance between the target and the substrate was 55 mm, while the base vacuum was kept to less than 2 × 10^–5^ Pa. The substrate was maintained at room temperature by a water cooling system. The sputtering chamber was filled with 8:1 Ar and N_2_ mixed gas, and the total pressure was maintained at 0.27 Pa. The DC sputtering current was 1.80 A, while the film was grown onto the substrate at a rate of 8 Å/s.

Finally, we immersed the NbN film on PI with a Si substrate into HF for approximately 15 min, and the NbN film with a PI layer completely peeled off from the Si substrate, as shown in Fig. 1d. A series of photographs of the flexible NbN film on PI with a diameter of 2 inches is shown in Fig. 1e–h. The film surface is flat and smooth, and the film can be bent at any angle. This method can not only precisely control the thickness of both the PI layer and the NbN film but can also be extended to larger areas and other flexible superconducting films. Since the film will curl naturally after peeling off from the Si substrate, which makes characterization by XRD and AFM difficult, we used samples with Si substrates for these kinds of measurements. Samples peeled off from Si substrates were used for the characterization of superconductivity with or without a magnetic field, unless otherwise indicated.

## Results and discussion

**Figure 3:**
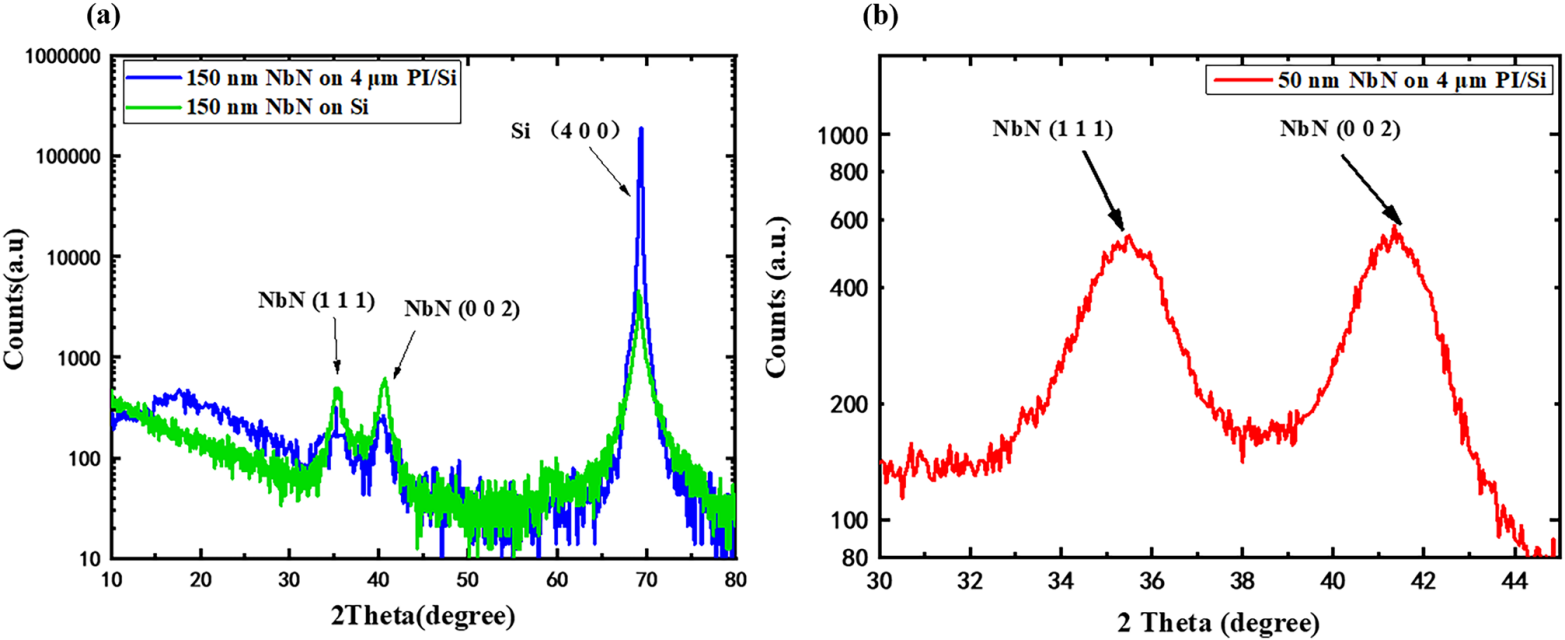
(**a**) XRD of 150 nm NbN on 4 μm PI with Si substrate and 150 nm NbN on Si substrate (**b**) GIXRD of 50 nm NbN on 4 μm PI/Si substrate.

**Figure 4:**
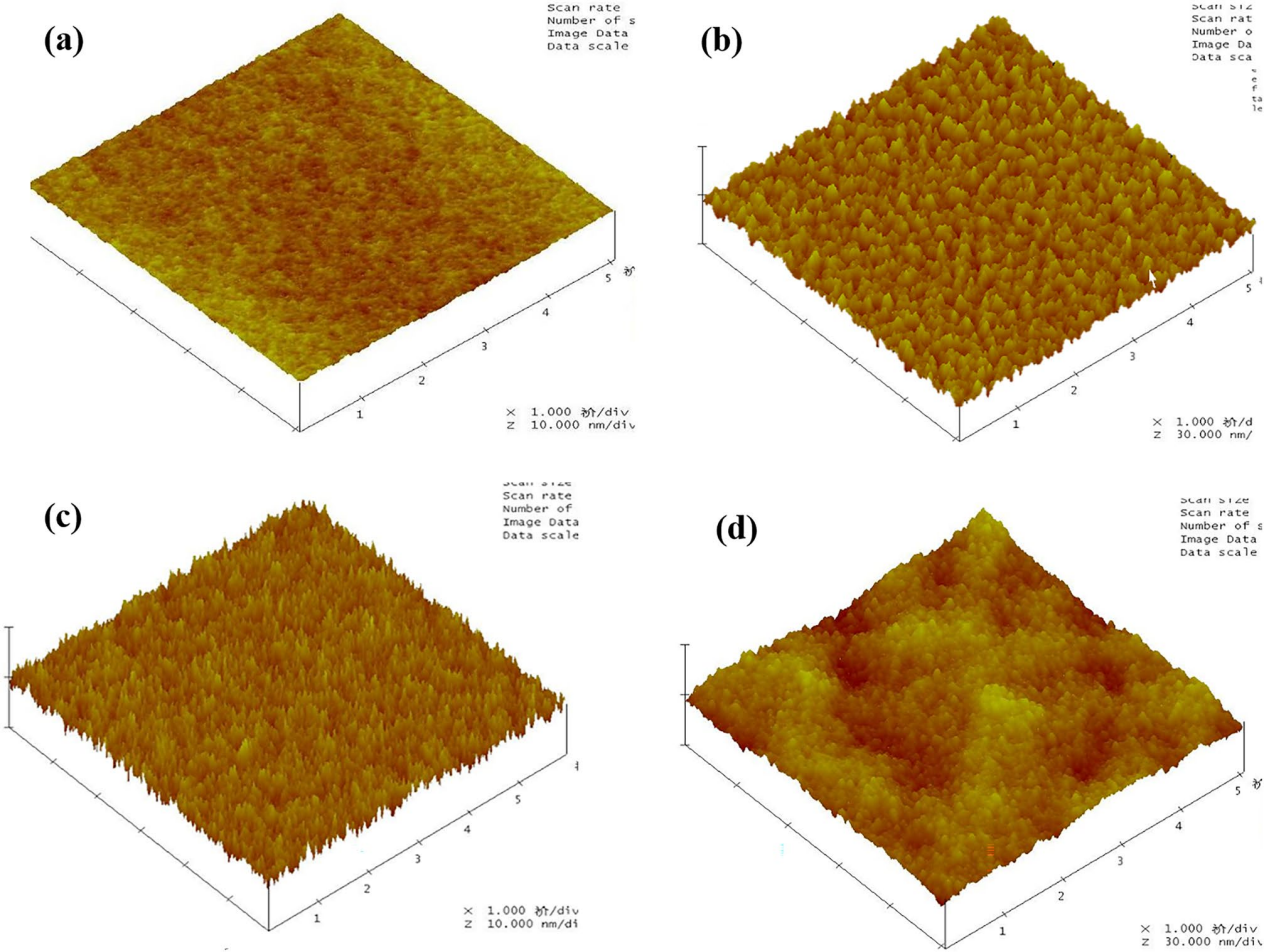
AFM image of NbN film on 4 μm PI with Si substrate: (**a**) without NbN film (**b**) 8 nm NbN film (**c**) 50 nm NbN film (**d**) 200 nm NbN film.

We used XRD to confirm the crystal structure of the NbN films on PI. As shown in Fig. 3a, a 150 nm NbN grown on a Si substrate is polycrystalline and shows a NbN (111) diffraction peak at 35.18°, and the NbN (002) diffraction peak is observed at 40.70°, which is similar to that for the film on 4 µm thick PI with a Si substrate having a NbN (111) diffraction peak at 35.20° and a NbN (002) peak at 40.52°. In addition to the different heights and positions of the diffraction peaks, the film on the PI substrate shows an obvious halo peak for the amorphous phase at approximately 20°, while the film on the Si substrate does not show this peak. Because the PI layer itself has no lattice structure, the source of the halo peak observed in XRD is most likely the NbN film. In addition, the NbN (111) and (002) diffraction peaks appear in the XRD pattern of this sample. This implies that an amorphous NbN layer is first grown onto the PI, and then the preferred orientation of the NbN film appears due to the self-buffering effect. To further confirm the lattice structure information for the NbN film on PI, we further used GIXRD with the 1 W/1 A beamline at the diffuse scattering station in the Beijing Synchrotron Radiation Facility (BSRF) to investigate a sample composed of a 50 nm thick NbN film on 4 μm PI with a Si substrate. By adjusting the grazing incidence angle, we can only obtain the lattice information for the film and exclude the influence of the substrate. The GIXRD images with grazing incidence depths of 10.5 nm are shown in Fig. 3b. Compared with Fig. 3a, even if the NbN film thickness is reduced to 1/3, obvious NbN (111) peaks at 35.45° and NbN (002) peaks at 41.35° are still observed. Considering that due to the setting of the grazing angle, the contribution of these diffraction peaks mainly arises from the 10.8 nm thick NbN film on top, which also implies that the thinner NbN film on PI will still have a preferred orientation. This is consistent with the results obtained for the superconducting properties of the films. AFM was used to characterize the flatness of both the PI layer and the NbN film surface in the area of 5 μm *5 μm. As shown in Fig. 4a, the root mean square (RMS) roughness of the 4 μm thick PI on the Si substrate is 0.183 nm, and the flatness is equivalent to that of the Si substrate, which meets the needs of growing NbN thin films. Figure 4b–d show that the RMS for NbN films with thicknesses of 8 nm, 50 nm, and 200 nm on 4 μm PI with Si substrates are 1.209 nm, 0.815 nm and 2.175 nm, respectively. The removal of the Si substrate will not affect the flatness of the film surface, which can lay the foundation for further application of flexible superconducting devices.

**Figure 5:**
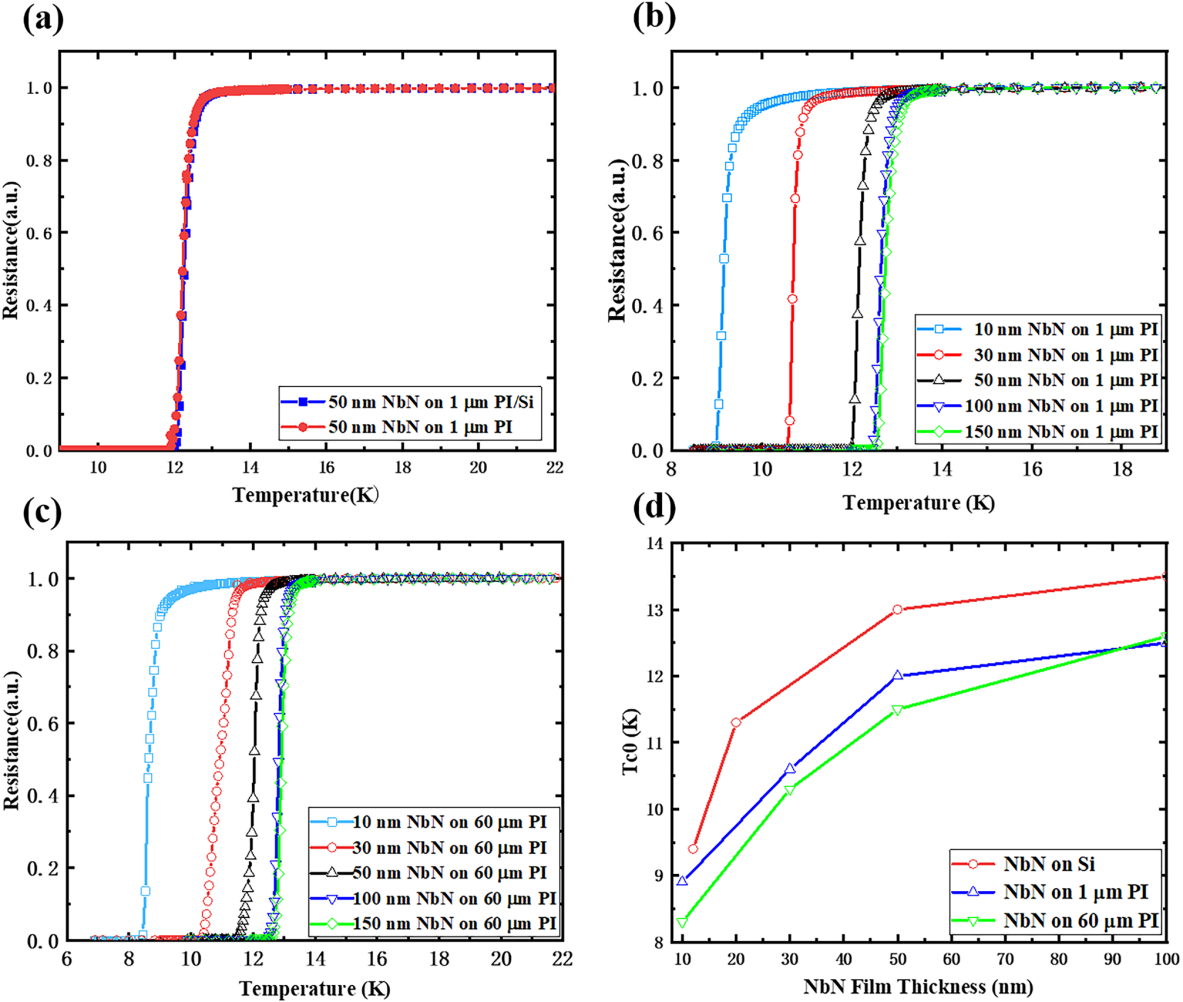
(**a**) R-T curves of 50 nm NbN films on 1 μm PI with and without Si substrate. (**b**) R-T curves of NbN films with different thicknesses on 1 μm PI. (**c**) R-T curves of NbN films with different thicknesses on 60 μm PI. (**d**) T_C0_ of NbN films with different thickness on PI and on Si substrates.

PPMS was applied to investigate the superconducting properties of the NbN films on PI. Figure 5a shows the R–T curves for 50 nm thick NbN films on 1 μm PI with and without a Si substrate. The two curves show near coincidence, the zero resistance transition temperature (T_C0_) difference between the two is approximately 0.1 K, and the results obtained for other samples with different thickness are similar. Therefore, removing the Si substrate has no significant adverse effect on the superconductivity of this flexible superconducting film. Figure 5b shows a series of resistance–temperature (R-T) curves for NbN films with different thicknesses on 1 μm PI. The T_C0_ for the 10 nm thick NbN film is 8.7 K, that for the 50 nm thick film is 11.9 K and that for the 150 nm thick film is 12.4 K. Figure 5c reveals the R–T curves for NbN with the same thickness at 60 μm PI. The T_C0_ for NbN films with thicknesses of 10 nm, 50 nm and 150 nm is 7.8 K, 11.4 K, and 12.5 K, respectively. It can be observed that the superconducting transition temperature slightly changes for the same thickness NbN film on PI layers of different thicknesses. The superconducting properties of flexible NbN films are comparable to those observed for NbN films on Si substrates^10^. Figure 5d summarizes the T_C0_ for different thickness NbN films prepared on the thickest (60 μm) and thinnest (1 μm) PI and compares them with the T_C0_ for NbN films grown directly onto Si substrates. The PI thickness has little effect on the superconducting transition temperature of NbN, while the T_C0_ of the NbN film grown directly onto Si is 1–2 K higher than that grown onto PI with the same thickness. A possible reason for this difference is that a layer of amorphous NbN film exists on the PI for self-buffering, which leads to a reduction of the effective thickness of the actual NbN film.

**Figure 6:**
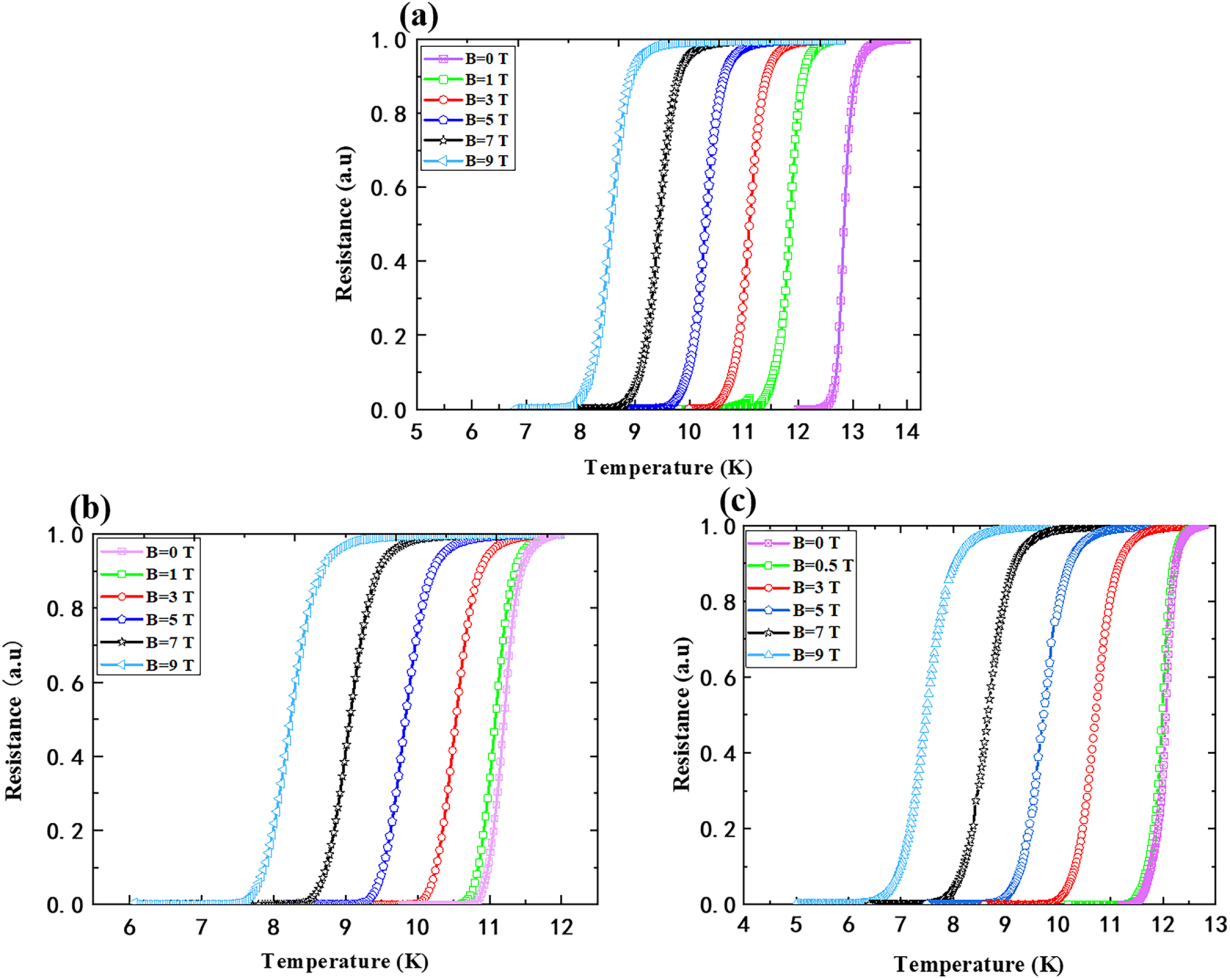
R-T curve of NbN films on 60 μm PI under different magnetic field: (**a**) 30 nm NbN (**b**) 50 nm NbN (**c**) 150 nm NbN.

For magnetic shielding applications of flexible NbN films, we also studied the superconductivity of the films under a magnetic field. The R-T curve for 30 nm thick NbN on 60 μm PI under different magnetic fields is shown in Fig. 6a. We can see that when there is no magnetic field, the T_C0_ for the film is 10.4 K, while it decreases to 7 K under a magnetic field of 9 Tesla (T). Figure 6b shows the R-T curves for 50 nm thick NbN on the same thickness PI under different magnetic fields. The T_C0_ for the film decreases from 11.4 K to 6.7 K when the magnetic field increases from 0 to 9 T. A similar situation for 150 nm NbN on 60 μm PI is also shown in Fig. 6c.

In addition, we also determined the upper critical magnetic field at different temperatures according to the R-T curves obtained for 30 nm, 50 nm and 150 nm thick NbN films on 60 µm PI under different magnetic fields. We fit the upper critical magnetic field of the film at 0 K, and the critical magnetic field is given by

1 $$H_{{{\text{c}}2}} (T) = H_{c2} (0)\left( {1 - \frac{T}{{T_{c} }}} \right)^{n}$$

**Figure 7:**
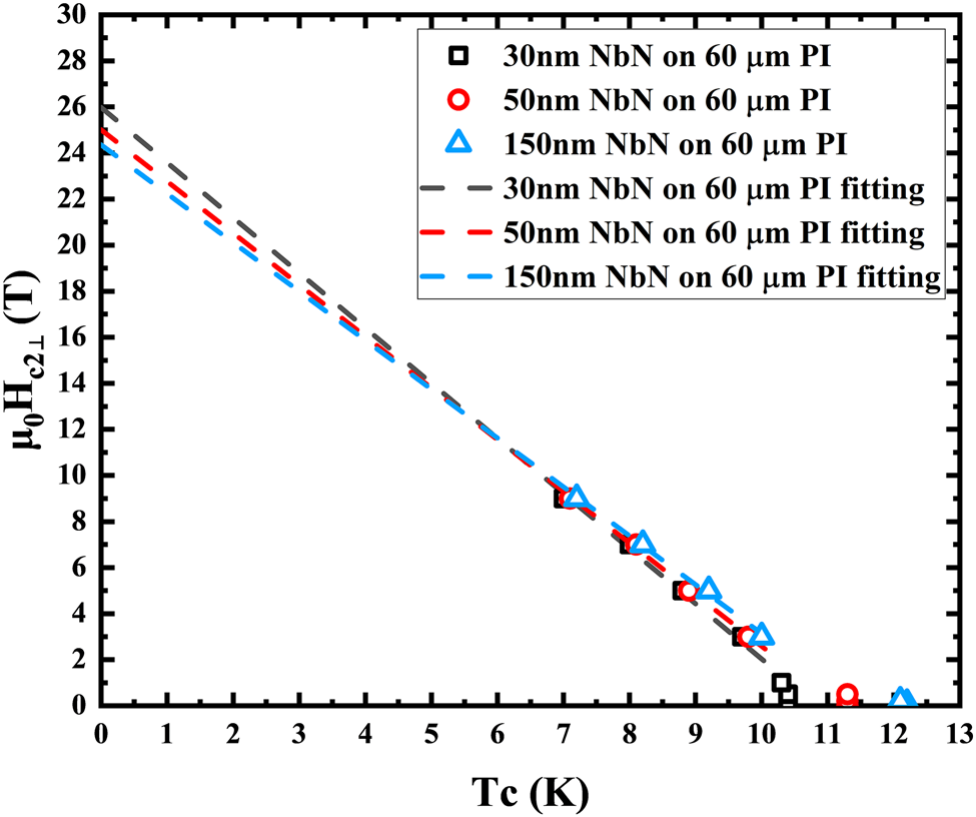
Superconducting upper critical magnetic field perpendicular to the NbN films on 60 μm PI measured and fitted with varied temperature.

For two-dimensional materials (d < ξ(T)), n≈0.5, and for three-dimensional materials (d ≥ ξ(T)), n≈1, where d represents the thickness of the film, and ξ(T) represents the coherence length of the films at temperature during the measurement^28, 29^. In our research, n = 1, since d ≥ ξ(T). Figure 7 shows that when the magnetic fields are perpendicular to the films, the upper critical magnetic fields for the 30 nm, 50 nm and 150 nm thick NbN are 25.97 T, 25.01 T, and 24.35 T, successively. Considering that the parallel upper critical magnetic field for three-dimensional materials is similar to the perpendicular upper critical magnetic field^30^, these data can be used to estimate the value for the parallel magnetic field. These results ensure the possibility of using this kind of flexible superconducting film in magnetic shielding applications.

## Conclusions

We have studied a method for preparing superconducting NbN films on flexible PI substrates with controllable thickness. Research into the structure and surface characteristics and superconducting properties of the films was carried out. The PI substrate and NbN films with different thicknesses on PI present high flatness, which is very important for the further preparation of devices. The flexible films exhibit superconducting properties similar to those obtained on Si substrates with or without a magnetic field. These flexible films can be further transferred to any substrate and adapted to different shape applications. In addition, they can be further processed into single-layer or multilayer flexible superconducting devices. This undoubtedly can further expand the application of NbN thin films.

## Data Availability

The datasets used and/or analyzed during the current study are available from the corresponding author on reasonable request.
